# Safety and efficacy of intravenous infusion of allogeneic cryopreserved mesenchymal stem cells for treatment of chronic kidney disease in cats: results of three sequential pilot studies

**DOI:** 10.1186/scrt198

**Published:** 2013-04-30

**Authors:** Jessica M Quimby, Tracy L Webb, Lauren M Habenicht, Steven W Dow

**Affiliations:** 1 Department of Clinical Sciences, Immunology, and Pathology, Center for Immune and Regenerative Medicine, College of Veterinary Medicine and Biomedical Sciences, Colorado State University, Fort Collins, CO 80523, USA; 2 Department of Microbiology, Immunology, and Pathology, Center for Immune and Regenerative Medicine, College of Veterinary Medicine and Biomedical Sciences, Colorado State University, Fort Collins, CO 80523, USA

**Keywords:** Cell culture, Cytokines, Feline, Kidney, Mesenchymal stem cells, Urine

## Abstract

**Introduction:**

Administration of mesenchymal stem cells (MSCs) has been shown to improve renal function in rodent models of chronic kidney disease (CKD), in part by reducing intrarenal inflammation and suppressing fibrosis. CKD in cats is characterized by tubulointerstitial inflammation and fibrosis, and thus treatment with MSCs might improve renal function and urinary markers of inflammation in this disease. Therefore, a series of pilot studies was conducted to assess the safety and efficacy of intravenous administration of allogeneic adipose-derived MSCs (aMSCs) in cats with naturally occurring CKD.

**Methods:**

Cats enrolled in these studies received an intravenous infusion of allogeneic aMSCs every 2 weeks collected from healthy, young, specific pathogen-free cats. Cats in pilot study 1 (six cats) received 2 × 10^6^ cryopreserved aMSCs per infusion, cats in pilot study 2 (five cats) received 4 × 10^6^ cryopreserved aMSCs per infusion, and cats in pilot study 3 (five cats) received 4 × 10^6^ aMSCs cultured from cryopreserved adipose. Serum biochemistry, complete blood count, urinalysis, urine protein, glomerular filtration rate, and urinary cytokine concentrations were monitored during the treatment period. Changes in clinical parameters were compared statistically by means of repeated measures analysis of variance (ANOVA) followed by Bonferroni’s correction.

**Results:**

Cats in pilot study 1 had few adverse effects from the aMSC infusions and there was a statistically significant decrease in serum creatinine concentrations during the study period, however the degree of decrease seems unlikely to be clinically relevant. Adverse effects of the aMSC infusion in cats in pilot study 2 included vomiting (2/5 cats) during infusion and increased respiratory rate and effort (4/5 cats). Cats in pilot study 3 did not experience any adverse side effects. Serum creatinine concentrations and glomerular filtration rates did not change significantly in cats in pilot studies 2 and 3.

**Conclusions:**

Administration of cryopreserved aMSCs was associated with significant adverse effects and no discernible clinically relevant improvement in renal functional parameters. Administration of aMSCs cultured from cryopreserved adipose was not associated with adverse effects, but was also not associated with improvement in renal functional parameters.

## Introduction

Chronic kidney disease (CKD) is a common condition in elderly cats and is characterized by tubulointerstitial inflammation, tubular atrophy and interstitial fibrosis with subsequent progressive loss of renal function [1,2]. Currently there is no definitive therapy short of renal transplant to improve kidney function in cats with CKD. Therefore, novel and effective therapeutic options are highly desired for treating this disease in cats.

Recently, a number of studies have suggested the use of mesenchymal stem cells (MSCs) as a novel treatment option for management of CKD, based on encouraging data from rodent studies [3-7]. There have been several rationales advanced for the use of MSCs for treatment of CKD. Rodent studies have suggested that MSCs may incorporate into the renal parenchyma and give rise to new renal tubular cells, though the process appears to be relatively inefficient [8-17]. MSCs also exert potent anti-inflammatory and antifibrotic effects and may therefore indirectly improve renal function by reducing disease-associated inflammation and fibrosis through paracrine effects [3-7]. For example, MSCs have been shown to inhibit lymphocyte proliferation and cytokine production, suppress dendritic cell function, and suppress interferon γ (IFNγ) production by natural killer (NK) cells [18].

Since inflammation appears to be present at all stages of CKD in cats, the immunomodulatory actions of MSCs are appealing as an alternative means of suppressing intrarenal inflammation long term and with fewer side effects than with conventional anti-inflammatory drugs. In the majority of experimentally-induced CKD models investigated, MSC administration resulted in improved renal function, decreased intrarenal inflammation, and reduction of renal fibrosis [3-7]. Thus, MSC therapy may be an effective new approach to slow the progression of CKD and improve renal function.

Previous studies have demonstrated that cultured MSCs can be administered intravenously to rodents as well as to humans. However, in rodent models there is a significant risk of pulmonary thrombosis when high numbers of MSCs are rapidly administered intravenously [19]. Alternative routes of delivery have included injection via the renal artery, injection directly into the renal parenchyma, and injection into the renal subcapsular space [3-7]. One potential advantage of intravenous delivery compared to other routes may be the induction of renotropic paracrine effects following intravenous administration of MSCs [20].

Based on compelling results from rodent studies, we conducted a series of pilot studies to investigate the safety and potential efficacy of adipose-derived MSC (aMSC) therapy as a treatment for cats with naturally occurring CKD. These studies were designed to test the hypothesis that allogeneic cryopreserved aMSCs could be safely administered to cats with CKD and would result in improvement in kidney function. For this study, aMSCs were derived from allogeneic, healthy, specific pathogen-free (SPF) donor cats and cryopreserved, either as cells or adipose tissue, prior to intravenous administration to study cats. One primary study endpoint was to determine safety and potential adverse effects of repeated intravenous administration of cryopreserved aMSCs. The second major endpoint was to determine whether repeated MSC administrations were associated with improvement in renal function or urinary markers of intrarenal inflammation. These studies produced evidence of modest, but unlikely to be clinically significant, improvement in renal function but also showed evidence of significant adverse effects associated with intravenous administration of higher doses of cryopreserved aMSCs.

## Methods

### Study cats

Cats with stable CKD, serum creatinine 1.6 to 5.0 mg/dl, were recruited from the patient population at the Veterinary Teaching Hospital at Colorado State University. Cats were determined to have stable CKD based on two repeated biochemical evaluations performed at least 2 weeks apart and ultrasonographic evidence of CKD. Pretreatment evaluation included complete blood count (CBC), biochemistry profile, urinalysis, urine culture, blood pressure, total T4, urine protein creatinine ratio (UPC), feline leukemia/feline immunodeficiency virus serology, and a renal ultrasound. Cats were excluded from the study if they had evidence of ureteroliths, pyelonephritis, anatomic abnormalities such as polycystic kidney disease or masses, uncontrolled hypertension, or concurrent systemic disease. Administration of concurrent supportive therapies was allowed provided there were no changes in therapy during the study period. The study was approved by the Institutional Animal Care and Use Committee at Colorado State University (#10-1603A and 11-2915A), and all owners reviewed and signed consent forms prior to participation in the study.

### aMSC preparation and administration: pilot studies 1 and 2

MSCs were isolated from adipose tissue of donor SPF cats obtained from the same subcutaneous site on the ventral abdomen just caudal to the umbilicus, as previously described [21,22]. For isolation of the stromal vascular fraction, the tissue was minced and digested with 1 mg/ml collagenase (Sigma Aldrich, St Louis, MO, USA) for 30 minutes at 37°C. The sample was centrifuged, and the stromal vascular fraction was plated in MSC medium (low glucose Dulbecco’s modified Eagle medium (DMEM), 100 U/ml penicillin, 100 μg/ml streptomycin, 2 μM l-glutamine, 1% essential amino acids without l-glutamine, 1% non-essential amino acids, 0.075% sodium bicarbonate (Invitrogen/Gibco, Carlsbad, CA, USA) plus 15% fetal bovine serum (Cell Generation, Fort Collins, CO, USA)). The aMSCs were incubated until approximately 70% confluent with media changes every 2 to 3 days. After reaching passage 3, cells were harvested, divided into treatment aliquots in freezing medium (11% dimethyl sulfoxide, 14% MSC medium, 75% fetal bovine serum), and stored in liquid nitrogen for no greater than 1 year prior to use. For injection in pilot studies 1 and 2, cryopreserved cells were removed from liquid nitrogen, incubated in MSC medium for 10 minutes, washed three times in Dulbecco’s phosphate buffered saline (DPBS; Sigma) and then counted and checked for viability using Trypan Blue (cells with less than 95% viability were not used). For cats in pilot study 1, 2 × 10^6^ cells were resuspended in 5 ml Hank’s balanced salt solution (HBSS) with 200 IU heparin sulfate and administered slowly by hand over 15 minutes; aMSCs were administered at weeks 0, 2 and 4. For cats in pilot study 2, 4 × 10^6^ cells were initially resuspended in 10 ml HBSS with 200 IU heparin sulfate and administered as a slow intravenous push over 30 minutes. However, the first cat to receive MSCs in this manner experienced an increased respiratory rate. Subsequently, cells for the high-dose group were resuspended in 20 ml HBSS/200 IU heparin and administered over 45 to 60 minutes with a syringe pump with frequent agitation of the syringe to prevent settling of the cells. Cats in pilot study 2 received aMSCs at weeks 2, 4, and 6. All three injections received by cats treated in pilot studies 1 and 2 were from the same donor cat. A total of four donor cats were used to provide aMSCs for those studies.

### MSC preparation and administration: pilot study 3

MSCs were isolated from adipose tissue of donor SPF cats as described above. For preparation of the adipose tissue for cryopreservation, the tissue was minced and divided into 1-g aliquots in 1 ml of freezing medium (11% dimethyl sulfoxide, 14% MSC medium, 75% fetal bovine serum), and stored in liquid nitrogen for no greater than 1 year prior to use. Cryopreserved adipose was then later thawed, immediately washed twice with DPBS, and then prepared for culture as described above. For injection in pilot study 3 cats, cells were harvested by trypsinization at passage 3 and resuspended in 10 ml DPBS with 200 IU heparin sulfate and administered as a slow intravenous push over 20 minutes. Cats in pilot study 3 received aMSCs at weeks 2, 4, and 6. All three injections received by cats treated in pilot study 3 were from different donor cats given in a prerandomized order. A total of three donor cats were used to provide aMSCs for the study.

### Characterization of MSCs

aMSCs cultured from cryopreserved adipose tissue were characterized by surface marker expression using flow cytometry and a panel of crossreactive antibodies specific for surface determinants expressed by MSCs from other species [23-26]. Specifically, feline aMSCs were analyzed for surface expression of CD44 (anti-mouse/human, antibody clone:IM7, eBioscience, San Diego, CA, USA) and CD90 (anti-human, antibody clone:eBio5E10, eBioscience). MSCs were also assessed for expression of CD4 (anti-feline antibody clone: 3-4F4, Southern Biotech, Birmingham, AL, USA) and major histocompatibility complex (MHC) class II (anti-feline antibody clone: TU39, BD Bioscience, San Jose, CA, USA). Samples were analyzed using a Cyan ADP flow cytometer (Beckman Coulter, Brea, CA, USA). Approximately 25,000 events were collected for analysis per sample.

*In vitro* differentiation assays were conducted to confirm the multipotency of feline aMSCs, as assessed by their ability to differentiate into three cell lineages (osteoblasts, chondrocytes, and adipocytes) that are characteristic of MSCs [27]. For differentiation into adipocytes, aMSCs at confluency were incubated with MSC medium supplemented with 0.5 μM dexamethasone (Sigma Aldrich), 50 μM indomethacin (Sigma Aldrich) and 0.5 μM 3-isobutyl-1-methylxantine (Sigma Aldrich) for 3 weeks with media changes every 3 to 4 days. Chondrogenic differentiation medium consisted of DMEM 1 × (Cellgro, Manassas, VA, USA) supplemented with 15% fetal bovine serum (FBS; Cell Generation), 10 nM dexamethasone (Sigma Aldrich), 10 ng/ml transforming growth factor (TGF)-β (R&D Systems, Minneapolis, MN, USA), 50 μg/ml ascorbic acid (Sigma Aldrich), and 40 μg/ml proline (Sigma Aldrich). Osteogenic differentiation medium consisted of MSC medium supplemented with 10 nM dexamethasone, 50 μM ascorbic acid, and 20 mM β-glycerophosphate (Sigma Aldrich) At the end of the differentiation period, cells were fixed with 10% neutral buffered formalin and stained with Oil Red O (Sigma Aldrich) for presence of lipid or with Alizarin Red (Sigma Aldrich) for the presence of calcium [27]. Cell pellets from cartilage differentiation were harvested and placed in OCT Compound (Sakura Finetek USA, Inc., Torrance, CA, USA) and flash frozen prior to staining with toluidine blue (Richard-Allan Scientific, Kalamazoo, MI, USA) for cartilage matrix. aMSCs cultured in MSC medium alone under identical conditions were used as differentiation controls.

### Clinical monitoring

#### Pilot study 1

Each treated cat underwent physical examination, weighing, and routine blood work consisting of CBC, serum biochemistry, urinalysis, and UPC immediately prior to aMSC injection at week 0, and at week 2, 4.

#### Pilot study 2

Each treated cat underwent physical examination, weighing, and routine blood work consisting of CBC, serum biochemistry, and urinalysis at weeks 0, 2, 4, 6 and 8. Each cat had a UPC performed at weeks 0 and 8. Additionally each cat had a glomerular filtration rate (GFR) estimated by iohexol clearance performed at weeks 0 and 8. Iohexol clearance has been described as a clinically applicable alternative to estimate GFR and has previously been assessed in cats with reduced renal function [28-30]. For this method, 300 mg/kg iohexol (Omnipaque, GE Health Incorporated, Princeton, NJ, USA) was administered intravenously, and blood samples were collected at 2, 3, and 4 h after administration. Analysis is commercially available at the Michigan State University Diagnostic Center for Population and Animal Health. Assessment of GFR variability was performed by enrolling three CKD cats that did not receive MSC therapy but underwent estimation of GFR by iohexol clearance at 0 and 8 weeks.

#### Pilot study 3

Each treated cat underwent physical examination, weighing, and routine blood work consisting of CBC, serum biochemistry, and urinalysis at weeks 0, 2, 4, 6 and 8. Each cat had a UPC performed at weeks 0 and 8. Additionally, each cat had a GFR by nuclear scintigraphy performed at weeks 0 and 8.

For the scintigraphy procedure, cats were sedated with a standard sedation protocol (ketamine 10 mg/cat and butorphanol 0.1 mg/kg intravenously once) at a standard time before the procedure. A single nuclear technician performed all of the procedures for each particular cat. For each procedure 1.0 mCi of Tc99m-labeled diethylene triamine penta-acetic acid (DTPA) (Cardinal Health, Dublin, OH, USA) was injected intravenously via a catheter placed in a standard location in each cat. Images were obtained using GE Millennium SPECT system applicable for small animal planar, whole body and single-photon emission computed tomography (SPECT) imaging (GE Healthcare, Waukesha, WI, USA). Three independent radiologists evaluated the GFR data, and a mean GFR value for each kidney as well as a global value was determined.

### Urinary cytokine analysis

Urine cytokine concentrations were determined using commercial ELISA kits, as we have described recently [31]. Briefly, urine concentrations of interleukin (IL)-6, IL-8, and IL-10 were measured using feline specific ELISA kits (R&D Systems), while monocyte chemoattractant protein 1 (MCP-1) and vascular endothelial growth factor (VEGF) were measured using commercial canine MCP-1 and VEGF kits respectively (R&D Systems). Urine transforming growth factor β1 (TGF-β1) concentrations were measured using an ELISA kit from Invitrogen (Camarillo, CA, USA).

Urine samples were collected by cystocentesis, and an aliquot of urine was immediately frozen at -20°C and then stored at -80°C for cytokine measurements. Samples were thawed immediately prior to analysis and kept on ice during sample preparation. All samples were centrifuged at 2000 rpm (350 *g*) for 5 minutes at 4°C, and the supernatant was collected for analysis. With one exception, ELISA kits were run according to manufacturer instructions for serum samples. As one exception, urine samples were diluted 5-fold rather than 40-fold for detection of TGF-β1.

Urine and serum creatinine concentrations were measured with a Roche Cobas Integra Chemistry Analyzer (Roche Diagnostics Limited, West Sussex, UK) at the Colorado State University Diagnostic Laboratory. All reported cytokine concentrations were normalized for differences in urine concentration as a urine cytokine-to-urine creatinine ratio. In order to maximize cytokine yield in the urine samples, additional sample processing steps were performed as previously described prior to running the IL-8, MCP-1, and VEGF ELISA kits [31].

### Statistical analysis

Changes in GFR, serum creatinine, blood urea nitrogen (BUN), electrolytes, urine protein-to-creatinine (UPC) ratio, urine specific gravity, and packed cell volume (PCV) data over time in the aMSC-injected cats were compared statistically by means of repeated measures analysis of variance (ANOVA) followed by Bonferroni’s correction. Values were considered statistically different for *P* <0.05. Statistical analyses were performed using Prism5 software (GraphPad, La Jolla, CA, USA).

## Results

### Description of study cats

A total of 165 cats with CKD were screened for entry into this series of pilot studies. In all, 52 cats were excluded for geographical reasons (extensive travel required), 40 cats were excluded due to concurrent illness, 18 cats were excluded due to end-stage disease, 13 cats were excluded as the owners thought the study too complex, 11 cats were excluded due to unstable disease, 8 cats were excluded for anatomic abnormalities, and 2 cats were excluded due to fractious attitude in hospital. Descriptive data for the 21 cats with CKD that were enrolled in the aMSC pilot studies are presented in Table 1. Two cats that were enrolled in pilot study 3 were unable to finish the trial due to development of unrelated illnesses. A summary of cat weights throughout the study and aMSC dose by weight are presented in Table 2. No statistically significant difference in weight was seen in any pilot study as determined by a Wilcoxon sign rank test.

**Table 1:** Summary of demographics of cats participating in the intravenous allogeneic cryopreserved studies

**Cat no.**	**Group**	**Description**	**IRIS CKD stage and creatinine (mg/dl)**	**Treatment**
1	Pilot study 1	10 yr MC DSH	IRIS CKD II; creatinine: 2.5	2 × 10^6 ^MSCs intravenously × 3 treatments
2	Pilot study 1	15 yr FS DSH	IRIS CKD III; creatinine: 3.5	2 × 10^6 ^MSCs intravenously × 3 treatments
3	Pilot study 1	7 yr MC Siamese	IRIS CKD III; creatinine: 4.3	2 × 10^6 ^MSCs intravenously × 3 treatments
4	Pilot study 1	12 yr MC DLH	IRIS CKD II; creatinine: 2.4	2 × 10^6 ^MSCs intravenously × 3 treatments
5	Pilot study 1	15 yr MC DSH	IRIS CKD II; creatinine: 2.3	2 × 10^6 ^MSCs intravenously × 3 treatments
6	Pilot study 1	15 yr FS Siamese	IRIS CKD III; creatinine: 3.5	2 × 10^6 ^MSCs intravenously × 3 treatments
7	Pilot study 2	11 yr MC DSH	IRIS CKD II; creatinine: 1.9	4 × 10^6 ^MSCs intravenously × 3 treatments
8	Pilot study 2	11 yr FS DSH	IRIS CKD II; creatinine: 2.6	4 × 10^6 ^MSCs intravenously × 3 treatments
9	Pilot study 2	18 yr FS DSH	IRIS CKD II; creatinine: 2.8	4 × 10^6 ^MSCs intravenously × 3 treatments
10	Pilot study 2	15 yr MC DSH	IRIS CKD II; creatinine: 2.2	4 × 10^6 ^MSCs intravenously × 3 treatments
11	Pilot study 2	7 yr MC DSH	IRIS CKD III; creatinine: 3.7	4 × 10^6 ^MSCs intravenously × 3 treatments
12	Pilot study 2	15 yr MC DSH	IRIS CKD II; creatinine: 2.3	Iohexol GFR repeatability only
13	Pilot study 2	16 yr MC DSH	IRIS CKD II; creatinine: 2.1	Iohexol GFR repeatability only
14	Pilot study 2	16 yr MC DSH	IRIS CKD II; creatinine: 1.8	Iohexol GFR repeatability only
15	Pilot study 3	9 yr MC DSH	IRIS CKD II; creatinine: 2.6	Enrolled but decompensated before treatment initiated
16	Pilot study 3	15 yr MC Siamese	IRIS CKD II; creatinine: 2.4	4 × 10^6 ^MSCs intravenously × 2 treatments before recurrence of previous diabetes
17	Pilot study 3	13 yr MC Siamese	IRIS CKD II; creatinine: 2.7	4 × 10^6 ^MSCs intravenously × 3 treatments
18	Pilot study 3	8 yr MC DSH	IRIS CKD II; creatinine: 1.7	4 × 10^6 ^MSCs intravenously × 3 treatments
19	Pilot study 3	13 yr MC DSH	IRIS CKD II; creatinine: 2.0	4 × 10^6 ^MSCs intravenously × 3 treatments
20	Pilot study 3	15 yr MC DLH	IRIS CKD II; creatinine: 2.3	4 × 10^6 ^MSCs intravenously × 3 treatments
21	Pilot study 3	15 yr FS DSH	IRIS CKD III; creatinine: 3.1	4 × 10^6 ^MSCs intravenously × 3 treatments

Six cats were enrolled in pilot study 1 and received 2 × 10^6^ cryopreserved adipose-derived mesenchymal stem cells (aMSCs) per infusion. In pilot study 2 eight cats were enrolled; five received 4 × 10^6 ^cryopreserved aMSCs per infusion, and three cats received only iohexol clearance studies. In pilot study 3, seven cats were enrolled; five cats received 4 × 10^6^ cryopreserved aMSCs cultured from cryopreserved adipose per infusion; two cats developed unrelated medical conditions and were removed from the trial. CKD, chronic kidney disease; GFR, glomerular filtration rate. MC = male castrated, FS = female spayed, DSH = domestic shorthair, DLH = domestic long hair, IRIS = International Renal Interest Society.

**Table 2:** Weight and mesenchymal stem cell (MSC) dose for cats participating in the intravenous allogeneic cryopreserved MSC studies

**Study**	**Stage**	**Weight in kg, median (range)**	**MSC dose in cells/kg, median (range)**
Pilot study 1, n = 6	Before	4.8 (2.7 to 5.1)	4.1 × 10^5 ^(3.9 × 10^5 ^to 7.4 × 10^5^)
	After	4.8 (2.5 to 5.2)	
Pilot study 2, n = 5	Before	4.8 (4.4 to 6.2)	8.3 × 10^5 ^(6.1 × 10^5 ^to 9.1 × 10^5^)
	After	4.8 (4.2 to 7.1)	
Pilot study 3, n = 5	Before	4.8 (3.7 to 7.1)	8.4 × 10^5 ^(5.6 × 10^5 ^to 1.1 × 10^6^)
	After	4.8 (3.5 to 7.1)	

### Characterization of MSCs

**Figure 1:**
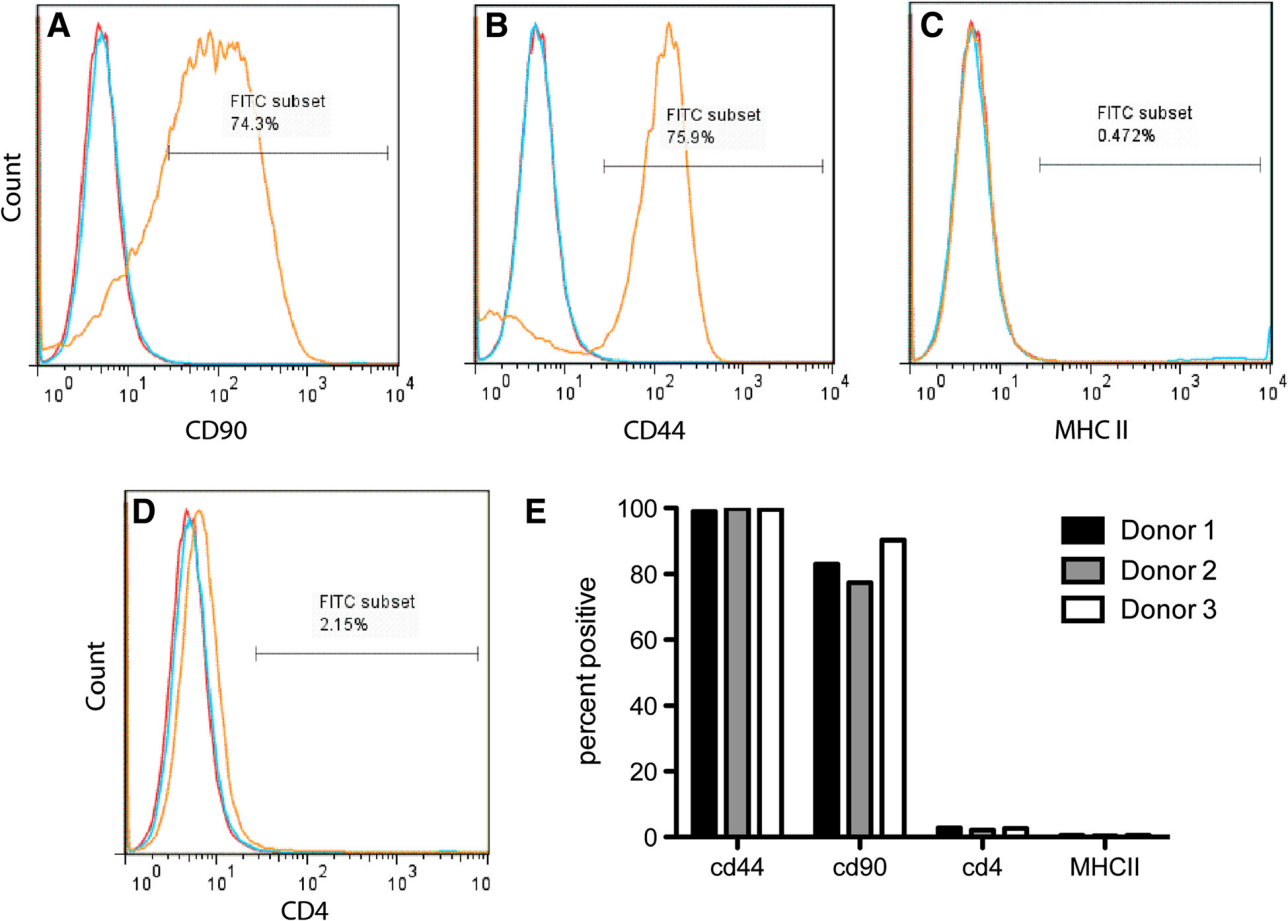
**Expression of cell surface markers by cryopreserved feline adipose-derived mesenchymal stem cells (aMSCs). **Feline aMSCs expanded from cryopreserved adipose were passaged three times in culture, then collected by trypsinization and immunostained for assessment of cell surface marker expression by flow cytometry, as described in Methods. Feline aMSCs expressed high surface levels of CD90 **(A)** and CD44 **(B) **but did not express major histocompatibility complex (MHC) class II **(C) **or CD4 **(D)**. Isotype controls are represented in red and unstained MSCs are represented in blue. Similar results were obtained with aMSCs from three donor cats **(E)**.

**Figure 2:**
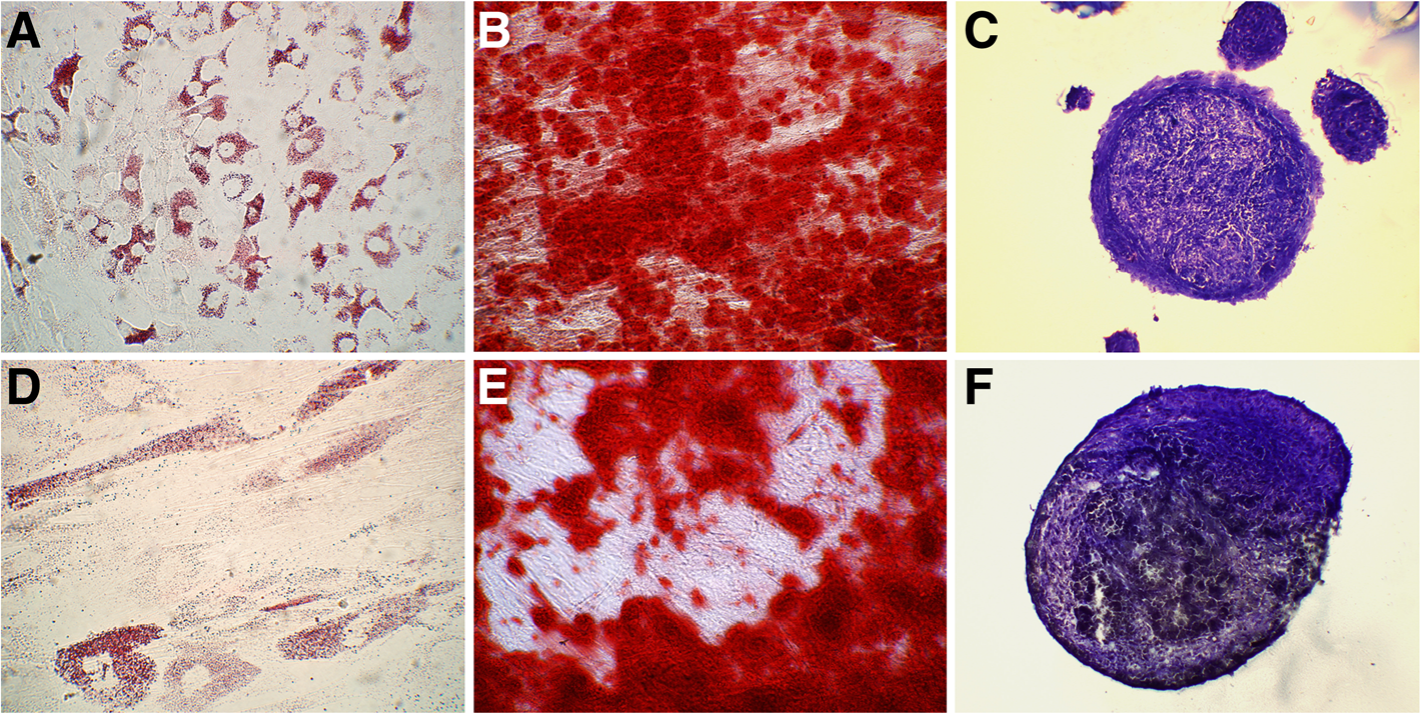
**Trilineage differentiation of feline adipose-derived mesenchymal stem cells (aMSCs) after cryopreservation as cells or adipose. **aMSCs taken directly from cryopreservation **(A-C) **and cultured from cryopreserved adipose **(D-F)** were capable of trilineage differentiation. (**A,D**) aMSCs formed intracellular lipid vacuoles when incubated in adipocytic differentiation media for 21 days. (B,E) aMSCs stained positive for calcium with alizarin red following differentiation into osteocytic phenotype after 21 days of incubation in differentiation media. (C,F) Cryosection of pellets of cartilage matrix (stained with toluidine blue) formed by aMSCs when exposed to chondrocytic differentiation media for 21 days.

During *in vitro* culture feline aMSCs were observed to develop into a relatively homogeneous population of plastic-adherent cells with fibroblast-like morphology. Adipose-derived MSCs expressed high levels of CD44 and CD90 and were negative for expression of CD4 and MHC class II (Figure 1). Both cryopreserved aMSCs and aMSCs cultured from cryopreserved fat were capable of trilineage differentiation (Figure 2).

### Short-term responses to MSC injection

Cats in pilot study 1 treated with three separate intravenous infusions of 2 × 10^6^ cryopreserved aMSCs and cats in pilot study 3 treated with three separate intravenous infusions of 4 × 10^6 ^aMSCs cultured from cryopreserved fat tolerated their injections without apparent adverse effects. During the infusions and immediately after, the cats appeared clinically normal and unperturbed. However, cats in pilot study 2, each having received an intravenous infusion of 4 × 10^6 ^cryopreserved aMSCs, experienced a number of treatment-related adverse effects. The adverse effects included vomiting (two cats) and increased respiratory rate (four cats). In two cats, vomiting and nausea occurred within 2 minutes of initiating the first aMSC infusion and were not associated with respiratory distress. Administration of diphenhydramine (1 mg/kg subcutaneously) and maropitant (1 mg/kg subcutaneously) resulted in cessation of clinical signs of vomiting and nausea. Affected cats were premedicated with diphenhydramine and maropitant for subsequent treatments, and additional adverse gastrointestinal reactions were not noted. One cat that experienced vomiting at the time of the first injection subsequently developed increased respiratory rate at the time of the third aMSC injection.

Increased respiratory rate and effort following aMSC infusion was not pronounced in three of the four pilot study 2 cats that experienced respiratory signs but were quite pronounced in one cat. Average baseline respiratory rate was 44 breaths/minute (range 36 to 60) and average increased respiratory rate was 80 breaths/minute (range 60 to 112 breaths/minute). Increased respiratory rate and effort was generally noted after approximately two-thirds of the aMSCs had been administered (that is, after 30 minutes of infusion) in the three cats with mild respiratory signs. One cat, however, had mildly increased respiratory rate on the first infusion, a normal second infusion, and developed marked overt respiratory distress on the third infusion after approximately one-third of the aMSCs had been administered. The respiratory distress was characterized by a sudden onset of increased respiratory rate that quickly led to nasal flare, open mouth breathing, and an orthopnic stance with head extended and elbows out. The injection was stopped, the cat was moved to a critical care ward and placed in an oxygen chamber, and diphenhydramine (1 mg/kg subcutaneously) was administered. After 20 minutes no improvement was noted, and the cat became notably nauseated (foaming at the mouth and subsequently vomiting). Maropitant (1 mg/kg subcutaneously) and dexamethasone (0.05 mg/kg intravenously) were administered. Clinical signs began to abate within 20 minutes of administration of dexamethasone. Open mouth breathing and orthopnea improved within 1 h. Increased respiratory effort continued for the next 8 h, but by 24 h post injection the cat was clinically normal and was discharged without further incident.

### Effects of MSC administration on renal function clinical parameters

**Figure 3:**
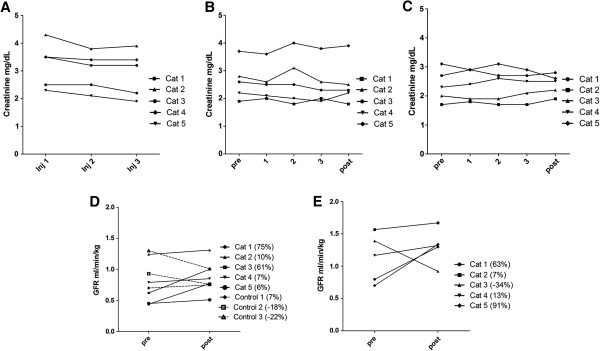
**Assessment of renal function in adipose-derived mesenchymal stem cell (aMSC)-treated cats. (A) **Serum creatinine values for cats in pilot study 1, who received three doses of 2 × 10^6 ^cryopreserved aMSCs intravenously 2 weeks apart. A statistically significant decrease in creatinine is seen (*P* = 0.01), however the degree of decrease may not be clinically significant. **(B)** Serum creatinine values for cats in pilot study 2, who received three doses of 4 × 10^6 ^cryopreserved aMSCs intravenously 2 weeks apart. No significant difference in creatinine was detected. **(C) **Serum creatinine values for cats in pilot study 3, who received three doses of 4 × 10^6^ aMSCs cultured from cryopreserved adipose intravenously 2 weeks apart. No significant difference in creatinine was detected. **(D) **Estimated glomerular filtration rate (GFR) by iohexol clearance results at 0 and 8 weeks for five cats in pilot study 2 that received 4 × 10^6 ^cryopreserved aMSCs intravenously every 2 weeks for three treatments. Control chronic kidney disease cats had GFR performed at 0 and 8 weeks only. **(E) **GFR determined by nuclear scintigraphy results at 0 and 8 weeks for five cats in pilot study 3 that received 4 × 10^6 ^aMSCs cultured from cryopreserved adipose intravenously every 2 weeks for three treatments.

Serum creatinine concentrations decreased significantly (*P* = 0.01) in pilot study 1 cats over the study period but were unchanged in cats in pilot studies 2 and 3 (Figure 3A-C), however it is our opinion that the degree of observed decrease (<0.5 mg/dl) in serum creatinine concentrations is of questionable clinical significance. It should be noted that one cat from pilot study 1 was removed from analysis of serum creatinine concentration because the owners failed to administer fluids during the week prior to assessment, in violation of the study protocol. Values for BUN, serum phosphorus, serum potassium, packed cell volume, and urine specific gravity or UPC did not change significantly following administration of aMSCs as compared to pretreatment values in any of the three pilot studies (Table 3).

**Table 3:** Summary of clinicopathological data from cats participating in the intravenous allogeneic cryopreserved mesenchymal stem cell (MSC) studies

**Study**	**Stage**	**Creatinine (mg/dl)**	**Blood urea nitrogen (mg/dl)**	**Phosphorus (mg/dl)**	**Potassium (meq/l)**	**Packed cell volume (%)**	**Urine protein-to-creatinine (UPC) ratio**	**Urine specific gravity**
Pilot study 1	Before	3.5 (2.3 to 4.3)	57 (33 to 69)	3.8 (2.6 to 4.5)	4.6 (3.8 to 5.5)	31 (29 to 36)	0.1 (0.1 to 0.2)	1.016 (1.013 to 1.022)
	After	3.2 (1.9 to 3.9)	53 (36 to 58)	3.7 (3.3 to 4.5)	4.2 (3.9 to 4.7)	28 (26 to 36)	0.1 (0.1 to 0.2)	1.016 (1.013 to 1.025)
Pilot study 2	Before	2.6 (1.9 to 3.7)	42 (34 to 61)	4.0 (3.6 to 4.2)	4.6 (3.7 to 5.1)	31 (27 to 35)	0.1 (0.1 to 0.2)	1.016 (1.015 to 1.030)
	After	2.3 (1.8 to 3.9)	37 (30 to 64)	4.7 (2.4 to 6.7)	4.6 (3.7 to 5.0)	29 (27 to 34)	0.1 (0.1 to 0.3)	1.017 (1.013 to 1.036)
Pilot study 3	Before	2.3 (1.7 to 3.1)	49 (34 to 53)	4.2 (3.6 to 4.9)	4.7 (3.9 to 5.0)	34 (25 to 37)	0.1 (0.1 to 0.4)	1.017 (1.013 to 1.037)
	After	2.5 (1.9 to 2.8)	42 (36 to 53)	3.8 (3.7 to 3.9)	4.7 (4.1 to 5.0)	33 (28 to 34)	0.1 (0.1 to 0.4)	1.019 (1.013 to 1.029)

No significant changes in clinicopathological parameters were seen as a result of repeated MSC administration in any pilot group.

### Effects of aMSC administration on estimated glomerular filtration rate

The GFR was not measured in pilot study 1 cats. In pilot study 2 cats, there was an overall trend towards improvement in estimated GFR by iohexol clearance values (*P* = 0.056), with individual increases of (75%, 10%, 61%, 7%, 6%) compared to untreated CKD control cats (7%, -18%, -22%) (Figure 3D). In pilot study 3 cats there was no statistically significant difference in total or individual kidney GFR values as determined by nuclear scintigraphy however variable individual increases were seen (63%, 7%, -34%, 13%, 91%) (Figure 3E).

### Effects of aMSC administration on urinary cytokine concentrations

**Figure 4:**
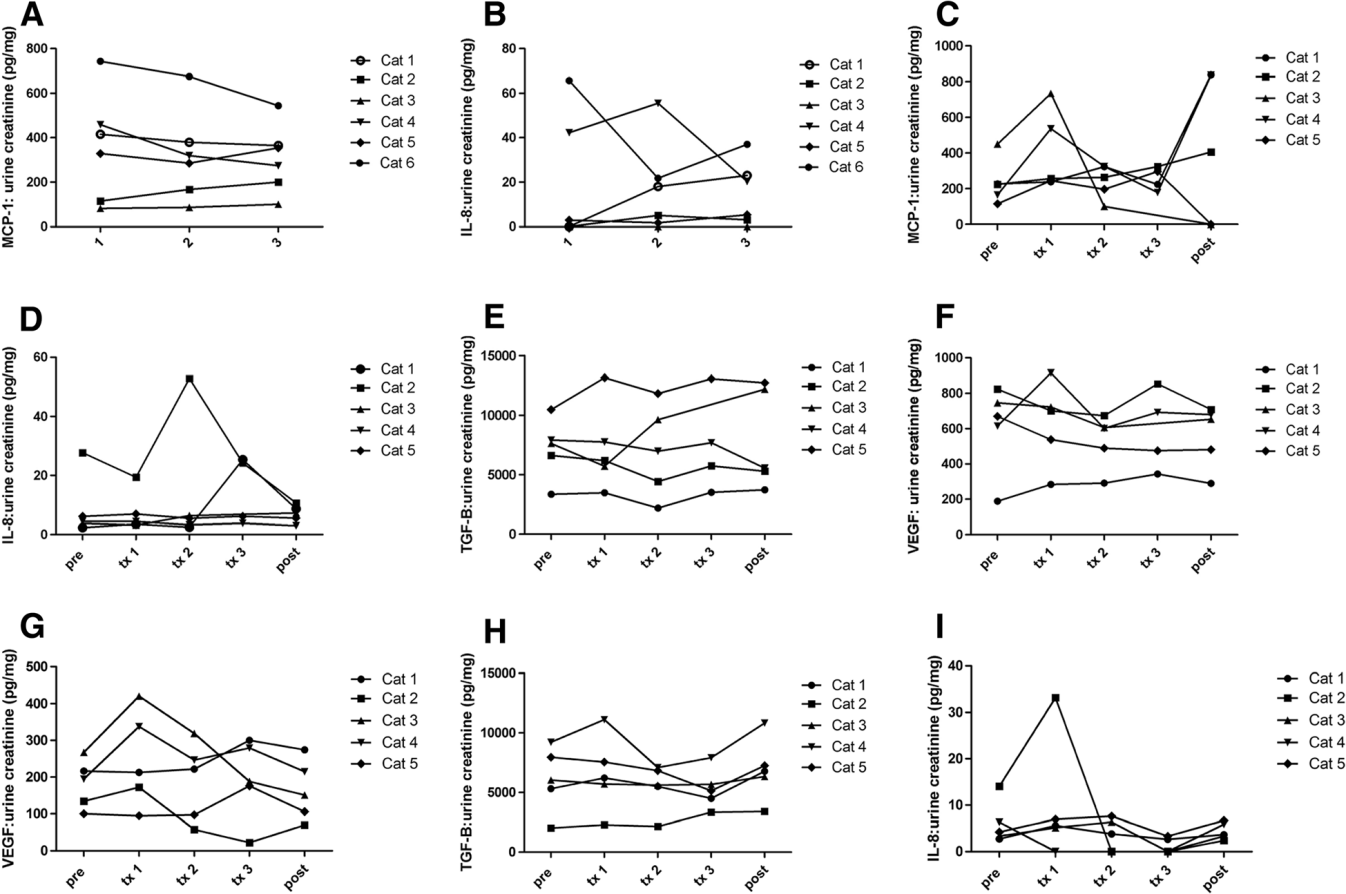
**Urinary cytokine levels in mesenchymal stem cell (MSC)-treated cats. **Pilot study 1 **(A,B)**: cats received 2 × 10^6 ^cryopreserved aMSCs intravenously every other week for three injections. (A) Monocyte chemoattractant protein 1 (MCP-1):urine creatinine ratio throughout the study. There was a statistically significant decrease in MCP-1 (*P *= 0.0001). (B) Interleukin 8 (IL-8):urine creatinine ratio throughout the study. There was a statistically significant decrease in IL-8 (*P *= 0.01). Pilot study 2 **(C-E)**: cats received 4 × 10^6 ^cryopreserved aMSCs intravenously every other week for three injections. Cytokine:urine creatinine ratio data throughout the study for (C) MCP-1, (D) IL-8, (E) transforming growth factor (TGF)-β1 and (F) vascular endothelial growth factor (VEGF). There was no statistically significant change in urinary cytokines in pilot study 2. Pilot study 3 **(G-I)**: cats received 4 × 10^6 ^aMSCs cultured from cryopreserved adipose intravenously every other week for three injections. Cytokine:urine creatinine ratio data throughout the study for (G) IL-8, (**H**) TGF-β1 and (**I**) VEGF. There was no statistically significant change in urinary cytokines in pilot study 3.

In cats in pilot study 1, urine MCP-1 and IL-8 concentration data following MSC administration as measured by ELISA are depicted in Figure 4A,B. Using repeated measures ANOVA, a statistically significant decrease in MCP-1 (*P* = 0.0001) as well as in IL-8 (*P* = 0.01) was detected. However due to the variability in cytokine concentration changes (some cats decreased significantly while others increased), these results may not have clinical relevance.

In cats in pilot studies 2 and 3, urine concentrations of IL-8, MCP-1, TGF-β1 and VEGF were assessed (Figure 2C-I). In the case of all four cytokines measured, repeated measures ANOVA failed to detect any significant changes in cytokine concentrations when pretreatment urine cytokine concentrations were compared to samples taken throughout the treatment period. In pilot study 3, MCP-1 levels were inconsistently detected in study samples.

## Discussion

The safety and potential efficacy of repeated intravenous administration of allogeneic cryopreserved feline aMSCs to cats with naturally occurring CKD was assessed in this series of pilot studies. MSCs have previously been shown to be effective in suppressing some aspects of renal disease in rodent models of induced CKD. However, MSC therapy has not been evaluated in a naturally occurring animal model of CKD such as the feline CKD model. We modified existing rodent MSC administration protocols to create a clinically feasible trial in cats with CKD with translational potential. The major findings from our preliminary studies of aMSC therapy were that lower doses of allogeneic cryopreserved aMSCs were well tolerated following repeated intravenous administration and appeared to be associated with modest improvement in renal function. The level of improvement in renal function seen in the treated cats is, however, of uncertain clinical significance. The six cats in pilot study 1 that received the 2 × 10^6^ cryopreserved aMSC dose experienced a statistically significant decrease in serum creatinine together with negligible adverse effects from aMSC administration. However, the degree of this decrease (<0.5 mg/dl) is very small and may not translate to any change in clinical disease. In contrast, higher intravenous doses of cryopreserved aMSCs were associated with a high incidence of adverse effects and variable evidence of improvement in renal function as evidenced by GFR estimated by iohexol clearance. Most of the five cats in pilot study 2 that received 4 × 10^6^ cryopreserved aMSCs per injection experienced side effects, including vomiting during infusion and increased respiratory rate and effort including overt dyspnea in one case. Significant changes in serum creatinine, GFR, and urinary cytokine concentrations were not observed in pilot study 2 cats. In pilot study 3, cats that received 4 × 10^6^ aMSCs cultured from cryopreserved adipose experienced no side effects, but little evidence of clinically significant improvement in renal function. Thus, we concluded that although intravenous administration of higher doses of cryopreserved, allogeneic aMSCs was unlikely to be an acceptable treatment option due to the high rate of complications and the lack of clinical or experimental responses; aMSCs expanded from cryopreserved fat appear a safer option although strong indication of efficacy is still lacking.

A recent study by Semedo *et al*. [4] in a rat model of CKD provided compelling evidence of the potential efficacy of repeated intravenous administration of bone marrow-derived MSCs for suppression of intrarenal inflammation and fibrosis, and this treatment protocol was the one we adapted for clinical application in CKD cats. Although aMSCs were used instead of bone marrow-derived MSCs, in the field of adipose stem cell research, the two sources are felt to be comparable in characterization and therapeutic potential in inflammatory disease states [23-27,32,33]. Why then were the results of our preliminary studies of aMSC therapy in cats with CKD so different from the rodent studies, when similar doses of MSCs (based on body weight) were utilized? Firstly, differences in the CKD model should be taken into consideration. Most rodent models involve acute insult to the kidney with subsequent administration of MSCs within a few weeks: a relatively short time post renal insult [4,34]. Thus, the induced disease models, with their relatively short time frame, may not be representative of the changes occurring in a truly chronic, naturally occurring disease process [11]. Cats have an extended lifespan and often have CKD for months to years prior to clinical diagnosis and study enrollment, which is more similar to the disease time frame seen in humans. The possibility that this potential therapy may not be as effective in patients with more long-standing disease should be considered.

Secondly, in our study, we utilized allogeneic aMSCs, whereas autologous MSCs were used in the Semedo *et al*. study [4]. The relative efficacy of autologous vs. allogeneic cells is an area of controversy. Although allogeneic MSCs are immune privileged and are not expected to incite an immune response, according to some authors they may not be as effective as autologous cells [35]. It is argued that autologous MSCs may survive longer in the body in comparison to allogeneic cells, which could reduce efficacy of the latter. Decreased efficacy of allogeneic MSCs in comparison to autologous MSCs has been observed in one acute renal failure rodent study [35]. However, allogeneic MSCs have been widely used in experimental stem cell transfer investigations, including clinical trials in humans, with positive results [35,36].

A third major difference was the use of freshly-thawed, cryopreserved aMSCs in pilot studies 1 and 2. This decision was made based on the logistical ease afforded by use of cryopreserved rather than freshly-cultured MSCs. Successful cryopreservation of cells has been previously described [37]. However, it is unknown if cryopreservation affects the functional properties of aMSCs necessary for successful use in this model. Previous studies assessing the effects of cryopreservation on MSCs did not examine effects on potential immunogenicity and further work is needed to fully assess this subject [37,38].

Based on the results of the three pilot studies, it appears that use of higher doses of cryopreserved aMSCs was the source of the treatment-related adverse effects in pilot study 2 as similar doses of aMSCs cultured from cryopreserved adipose tissue did not result in any adverse effects. Although the specific reasons for the increased incidence of side effects is not known, it is likely related to the increase in dose of cryopreserved cells. The most likely explanation for this reaction is an instant blood mediated inflammatory reaction (IBMIR) which results in clumping of the cells as they contact the blood and potential subsequent micro pulmonary thromboembolism [39]. The IBMIR phenomenon has been described previously in cryopreserved cells and increases in severity with dose and passage number [39]. It can result in lysis of the administered MSCs and subsequent poor efficacy. Although all cells given in pilot study 2 were of the same passage (P3) as those used in the other two pilot studies, the reaction was only seen in the pilot study group where cells were taken directly from cryopreservation and used at the higher dose. In pilot study 3 no complications during or after administration of aMSCs cultured from cryopreserved fat were appreciated. Thus, we have concluded that the administration of a higher dose of aMSCs taken directly from cryopreservation, despite careful washing, was the source of the toxic reactions observed, and this form of administration is not recommended.

Although several cats in the pilot study 1 group experienced a statistically significant improvement in serum creatinine concentrations during the study, the degree of increase is unlikely to be clinically significant. Creatinine is not the most sensitive measurement of renal function even though it is the routinely measured clinical marker of renal function. Several factors that are not directly related to renal function can influence serum creatinine values, including muscle mass and hydration status [28,40]. Decreases in weight and/or muscle mass can potentially decrease serum creatinine, while a worsening in hydration status can increase serum creatinine. In addition, changes to renal function may occur without any concomitant alteration in serum creatinine, although this is thought to be less true once an animal reaches the stage of moderate renal dysfunction [28]. Although there were no significant changes in body weight noted in these pilot studies, individual changes in body weight in the study cats could potentially have confounded serum creatinine measurements, while changes in bladder fullness at the time of weighing could potentially have compromised weight values. Therefore, in cats in pilot studies 2 and 3 we also measured estimated GFR, as a more objective way to monitor changes in renal function. Quantitating GFR allowed us to assess potential overall kidney function changes induced by the paracrine and/or autocrine effects of MSC administration. GFR results were variable in cats in pilot studies 2 and 3; two of five animals in pilot study 1 and two of five cats in pilot study 3 did experience a marked increase from pretreatment baseline; however the change in GFR was not significant overall for the group. Overall, definitive evidence of significant efficacy of intravenous administration of allogeneic aMSCs for treatment of CKD in cats is lacking at this time. Closer evaluation of the mechanism of MSC action in CKD, effect of disease stage, necessary cell dosage (for example, a cell/kg dose rather than a per cat dose as used in these pilot studies), timing of injections, and other variables remain to be determined.

## Conclusions

The safety and potential efficacy of repeated intravenous administration of allogeneic cryopreserved feline aMSCs to cats with naturally occurring CKD was assessed in this preliminary study. The major findings from our pilot studies of aMSC therapy in cats with CKD were that lower doses of allogeneic cryopreserved aMSCs were well tolerated following repeated intravenous administration and appeared to be associated with modest decrease in serum creatinine. In contrast, higher intravenous doses of cryopreserved aMSCs were associated with a high incidence of adverse effects and only modest evidence of functional improvement in renal function. Thus, we concluded that intravenous administration of higher doses of cryopreserved, allogeneic aMSCs was unlikely to be an acceptable treatment option due to the high rate of complications and the lack of clinical or experimental responses.

## Abbreviations

aMSCs: Adipose-derived mesenchymal stem cells; CBC: Complete blood count; CKD: Chronic kidney disease; GFR: Glomerular filtration rate; HBSS: Hank’s balanced salt solution; IBMIR: Instant blood mediated inflammatory reaction; IL: interleukin; MCP-1: Monocyte chemoattractant protein 1; MSCs: Mesenchymal stem cells; SPF: Specific pathogen-free; TGF-β1: Transforming growth factor-β1; UPC: Urine protein-creatinine ratio; VEGF: Vascular endothelial growth factor.

## Competing interests

The authors declare they have no competing interests.

## Authors’ contributions

JMQ conceived and designed the study, harvested adipose tissue, prepared injections, provided veterinary care and administered treatments to enrolled animals, performed data acquisition, analysis and manuscript writing and revision. TLW performed the isolation and expansion of MSCs, urine cytokine analysis, flow cytometry and differentiation assays, prepared injections, and manuscript writing and revision. LAH performed the sample preparation, data entry, and urine cytokine analysis, and manuscript writing. The study was conducted in the laboratory of SWD and he was responsible for supervision of the study and manuscript writing and revision. All authors read and approved the final manuscript.
